# Extracellular HtrA2 Induces Apoptosis in Human Umbilical Vein Endothelial Cells

**DOI:** 10.3390/ijms20215446

**Published:** 2019-10-31

**Authors:** Gurpinder Kaur, Daniela Stallmann, Nancy Schanze, Rosmarie Laumann, Lukas Andreas Heger, Johannes Steinfurt, Peter Stachon, Karlheinz Peter, Christoph Bode, Martin Moser, Ingo Ahrens, Daniel Duerschmied, Marcus Hortmann

**Affiliations:** 1 Department of Cardiology and Angiology I, Heart Center Freiburg University, Faculty of Medicine, University of Freiburg, 79106 Freiburg, Germany; gurpinder.kaur@universitaets-herzzentrum.de (G.K.); daniela.stallmann@universitaets-herzzentrum.de (D.S.); nancy.schanze@universitaets-herzzentrum.de (N.S.); rosmarie.laumann@uniklinik-freiburg.de (R.L.); lukas.heger@uniklinik-freiburg.de (L.A.H.); johannes.steinfurt@universitaets-herzzentrum.de (J.S.); peter.stachon@universitaets-herzzentrum.de (P.S.); christoph.bode@universitaets-herzzentrum.de (C.B.); martin.moser@universitaets-herzzentrum.de (M.M.); daniel.duerschmied@universitaets-herzzentrum.de (D.D.); 2 Baker Heart and Diabetes Institute, PO Box 6492, Melbourne, VI 3004, Australia; Karlheinz.Peter@baker.edu.au; 3 Department of Cardiology, Augustinerinnen Hospital, Academic Teaching Hospital, University of Cologne, 50678 Cologne, Germany; IAhrens@severinskloesterchen.de

**Keywords:** HtrA2 (high temperature required protein A2), ischemia reperfusion injury, apoptosis

## Abstract

The serine protease high-temperature-required protein A2 (HtrA2) has been identified as a key intracellular molecule promoting apoptosis in cells during ischemia reperfusion (IR) injury. IR injury in ST-segment elevation myocardial infarction (STEMI) contributes to overall myocardial damage. HtrA2 has further been shown to be significantly increased in the serum of patients with STEMI. In the present pilot study, we use human umbilical vein endothelial cells (HUVECs) to investigate whether extracellular HtrA2 induces apoptosis using Annexin V staining. Furthermore, we examine whether HtrA2 is released extracellularly after staurosporine-induced apoptosis using ELISA. We find that HtrA2 is released upon induction of apoptosis by staurosporine into the cell culture medium. Furthermore, treatment of HUVECs with extracellular HtrA2-induces apoptosis, while the addition of anti-HtrA2 antibodies reduces both HtrA2- and staurosporine-induced endothelial cell apoptosis. In conclusion, we show here that extracellular HtrA2 induces apoptosis in human endothelial cells, although the exact molecular mechanisms have to be investigated in future.

## 1. Introduction

According to the World Health Organization’s report of 2016 ischemic heart disease is the major cause of death globally [1]. To prevent the death of cardiomyocytes caused by myocardial infarction, early intervention against ischemia is extremely important, using either thrombolytic therapy or percutaneous coronary intervention for reperfusion [2]. However, as a consequence of the reinitiated blood flow further cardiomyocyte death is induced by ischemia reperfusion (IR) injury. IR injury causes rapid fluctuation of pH, oxidative stress, intracellular (Ca^2+^) overload and infiltration of inflammatory neutrophils, generating reactive oxygen species, further causing endothelial dysfunction [3,4,5,6,7,8,9].

During IR injury, cardiac cell death is mediated by different mechanisms [10,11]. Necrosis as a passive mechanism causes unregulated cell death and a loss of plasma membrane integrity. Necrosis is primarily involved in ischemia, whereas apoptosis has been described as the main mechanism in reperfusion injury in a murine model of acute myocardial infarction [12]. Hence, the quantitative contribution of apoptosis and necrosis to the death of cardiomyocytes remains unclear. Mitochondria play a pivotal role in apoptosis induction, as Bcl-2 proteins and calcium overload promote mitochondrial membrane permeabilization and cause the release of apoptotic factors into the cytosol [6,7,8,13].

The high-temperature-required protein A2 (HtrA2) is an apoptosis-inducing protein that is released after an apoptotic stimulus from the mitochondrial intermembrane space into the cytosol, where it inhibits the inhibitors-of-apoptosis proteins (IAPs). Previous studies have shown that HtrA2 is released after IR injury from the mitochondrial intermembrane space of cardiomyocytes together with cytochrome c to the cytosol, where it finally leads to apoptosis via caspase activation [14]. Treatment with the HtrA2 inhibitor UCF-101 ameliorated infarct size in a rat model of myocardial ischemia reperfusion injury [15]. In addition, in aging rat hearts the expression and leakage of HtrA2 is increased and IR injury is enhanced [16]. Furthermore, it has been reported that overexpression of HtrA2 in a mouse model promotes cardiomyocyte apoptosis and leads to cardiac dysfunction in vivo [17].

In a recent study, HtrA2 has been shown to be significantly increased in serum of patients with ST-segment elevation myocardial infarction (STEMI) and shows promise as a potential biomarker for IR-injury. [18]. 

Cytochrome c has been shown to be released together with HtrA2 into the cytosol; additionally, for cytochrome c it has been demonstrated that it is released into the extracellular space [19,20,21]. To our knowledge, it is not known whether HtrA2 is released extracellularly after apoptosis induction or whether it contributes extracellularly to induction of apoptosis. If it is validated that extracellular HtrA2 might have any impact on cell death, it may present a specific extracellular target for pharmacological cardio protection strategies.

The aim of this study is to investigate whether HtrA2 is detectable in the extracellular space during apoptotic cell death. To further analyze and determine future inhibitory targets for apoptotic cell death, we assess if extracellular HtrA2 induces apoptosis in human umbilical vein endothelial cells (HUVEC).

## 2. Results

### 2.1. Release of HtrA2 into the Extracellular Space

**Figure 1:**
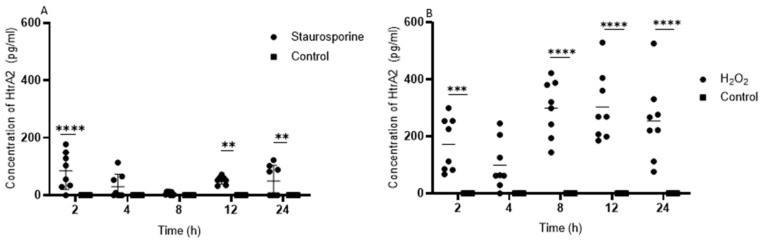
(**A**) High-temperature-required protein A2 (HtrA2) is found after staurosporine induction of apoptosis in the supernatant of human umbilical vein endothelial cells (HUVECs). (**B**) HtrA2 is significantly increased in the supernatant of HUVECs after H_2_O_2_-induced necrosis. Data is presented as dot plots with means and Standard Deviation(S.D). Significantly different vs. corresponding control with **** *p* < 0.0001, *** *p* <0.001 and ** *p* < 0.01.

Staurosporine induction of apoptosis significantly increased HtrA2 in the culture medium of HUVECs (Figure 1A), whereas HtrA2 was absent after vehicle treatment at every time point. Interestingly, while up to 4 h of incubation HtrA2 was increased compared to the control, at time point 8 h virtually no HtrA2 was detectable. This was followed by a second HtrA2 increase after twelve hours of stimulation. After H_2_O_2_ exposure, significant amounts of HtrA2 were detectable in the supernatant of HUVECs at each time point (Figure 1B). Higher HtrA2 concentrations were measured after H_2_O_2_ induction of necrosis compared to staurosporine treated cells.

### 2.2. Extracellular Effects on HUVEC

**Figure 2:**
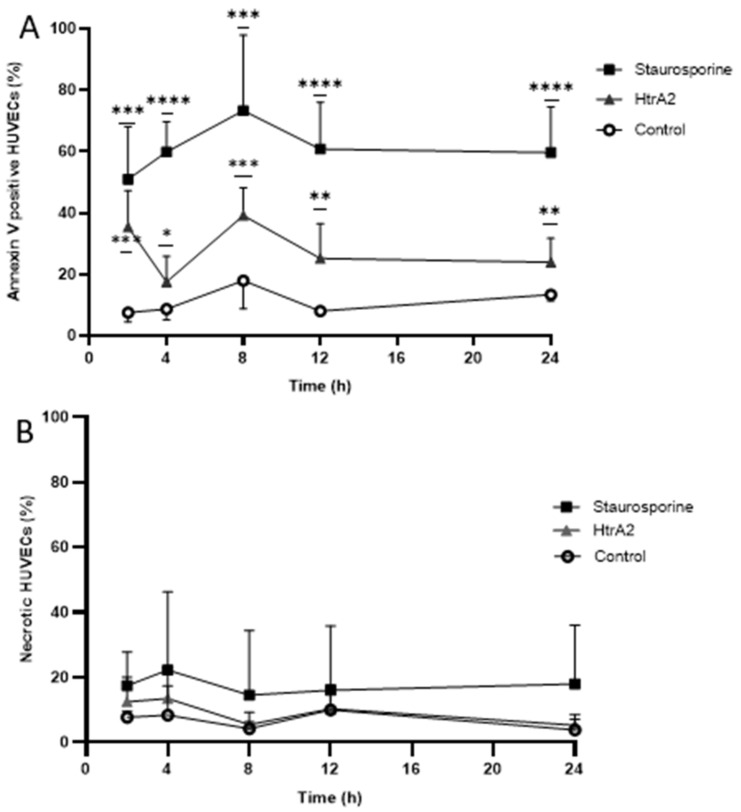
Extracellular HtrA2-induced apoptosis (**A**) but not necrosis (**B**) in HUVECs. HUVEC were stimulated with (2 µg/mL) of recombinant HtrA2, staurosporine (200 nM) or vehicle for a 24-h period. Results are presented as means with S.D. Significantly different compared to corresponding control with **** *p* < 0.0001 *** *p* < 0.001, ** *p* < 0.01 and * *p* < 0.05.

**Figure 3:**
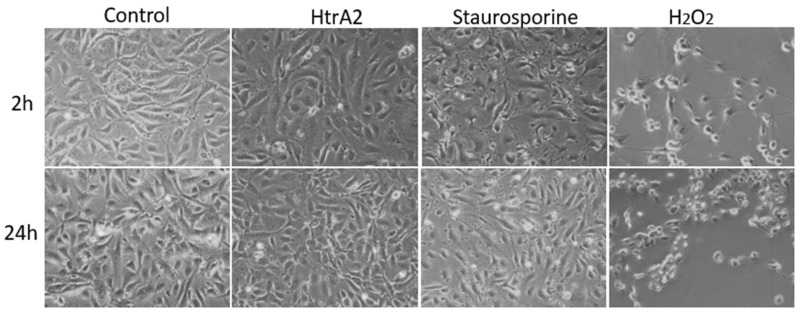
Morphology of HUVEC treated with HtrA2, staurosporine, H_2_O_2_ or untreated control. Morphology is visualised under microscope (magnification, 40×).

To assess whether extracellular HtrA2 induces apoptosis in HUVECs, we added HtrA2 (2µg/mL) to the culture medium of HUVECs over a 24 h time course. Extracellular HtrA2 significantly induced apoptosis in HUVECs compared to vehicle control over the whole indicated duration of time (Figure 2A). Next, we analyzed whether extracellular HtrA2 may have a necrotic effect in human endothelial cells. The propidium iodide (PI) staining demonstrated that extracellular HtrA2 did not induce necrosis for up to 24 h at the applied concentration (Figure 2B). Furthermore, staurosporine (used as a selectively apoptosis inducing positive control) induction led to significant apoptosis, whereas it did not contribute significantly to necrosis in HUVECs (Figure 2B). As expected, H_2_O_2_ caused a marked change in cell morphology, indicating necrosis (Figure 3). Staurosporine-treated cells showed morphological characteristics of apoptosis such as shrinkage and irregular shape. According to the lower degree of apoptosis induction by HtrA2 compared to staurosporine (Figure 2), no gross changes in morphology were observed after treatment with HtrA2 (Figure 3).

**Figure 4:**
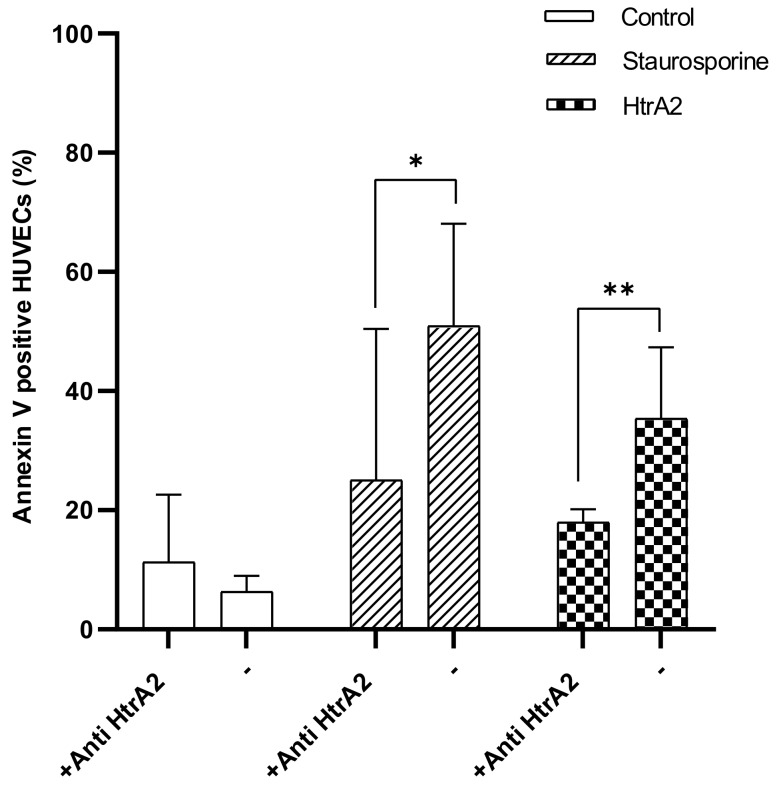
Anti-HtrA2 antibodies partially inhibited extracellular HtrA2 and staurosporine-induced apoptosis. HUVEC cultures were incubated in n the presence and absence of staurosporine (200 nM), HtrA2 (2 μg/mL) and anti-HtrA2 (2 μg/mL). The percentage of apoptotic HUVECs was determined after 2 h by Annexin V staining. Results are presented as means with S.D that are significantly different to corresponding control with ** *p* <0.01 and * *p* < 0.05.

**Figure A1:**
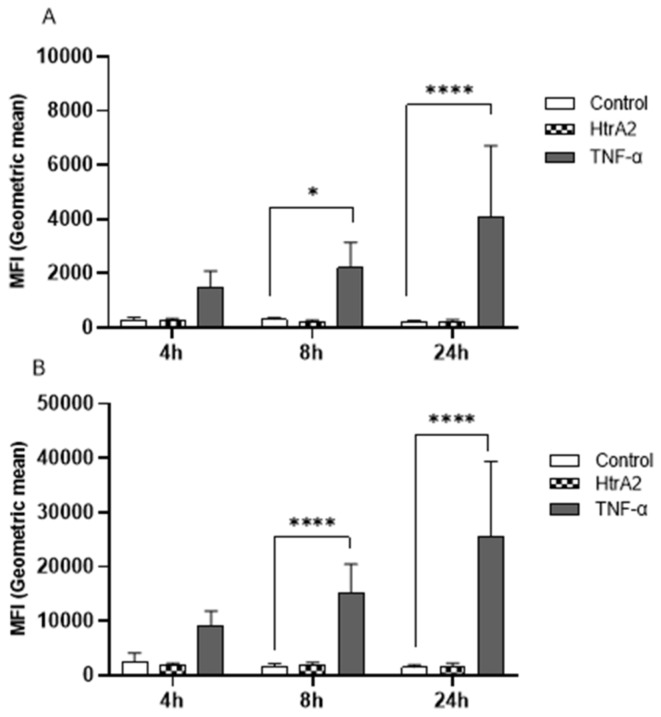
Extracellular HtrA2 did not activate ICAM and VCAM. HUVEC cultures were incubated in HUVEC medium in the presence and absence of TNFα (10 ng), HtrA2 (2 μg/mL) and HtrA2+ TNFα. The expression of VCAM (**A**) and ICAM (**B**) in HUVECs was determined after 4-, 8- and 24h h by measuring the mean fluorescence intensity (MFI). Results are means with S.D. Significantly different with **** *p* < 0.0001 and * *p* < 0.05.

Next, we investigated whether anti-HtrA2 antibodies diminished extracellular HtrA2 and staurosporine-induced apoptosis. Anti-HtrA2 antibodies were added concomitantly with the induction of staurosporine and extracellular HtrA2 apoptosis to the culture medium of HUVECs. The anti-HtrA2 antibody diminished staurosporine (Figure 4) and extracellular HtrA2 (Figure 4)-induced apoptosis significantly in HUVECs after 2 h of incubation. Furthermore, we investigated whether extracellular HtrA2 led to an upregulation of vascular adhesion molecule (VCAM) and intracellular adhesion molecule (ICAM). Extracellular HtrA2 did not induce upregulation of neither VCAM or ICAM (Figure A1). 

## 3. Discussion

In this study, we investigated for the first time whether HtrA2 is detectable in the extracellular space of HUVECs after apoptosis induction and whether extracellular HtrA2 induces apoptosis in HUVECs.

Apoptosis plays an important role in the pathogenesis of a variety of cardiovascular diseases [22]. Notably, the apoptosis pathway contributes in part to myocardial IR injury [23]. Although there is rapid progress in the treatment of myocardial ischemia, more than one third of STEMI patients do not show complete reperfusion due to severe IR injury [24,25]. Growing evidence indicates that HtrA2, a pro-apoptotic protein, is associated with myocardial reperfusion injury [14,15,16,17,18,26] and that targeted anti-apoptotic treatments may improve clinical outcomes in patients with ischemic heart disease [27].

In our study, we report four major findings: first we found a significantly increased extracellular concentration of HtrA2 after apoptosis induction compared to untreated cells.

Furthermore, we described that extracellular HtrA2 causes apoptosis in HUVECs, which is diminished using an anti-HtrA2 antibody. Interestingly, anti-HtrA2 antibodies also reduced staurosporine-induced apoptosis.

A key regulator of the apoptotic pathway and well-known protein is cytochrome c. The release of cytochrome c after apoptosis from the mitochondria into the cytosol and from there into the extracellular space has been proposed by several authors so far [19,20,21]. However, the release of HtrA2 after apoptosis into the extracellular space has not been demonstrated to our knowledge.

In this study, we demonstrated for the first time in vitro that HtrA2 is released not only into the cytoplasm but moreover leaves the cell after apoptosis induction. Understandably, HtrA2 was significantly detected in the extracellular space after necrosis induction. Necrosis causes plasma membrane rupture, causing all the cell content, including intracellular HtrA2, to enter the extracellular space.

It has been shown that IR injury leads to the mitochondrial release of HtrA2 into the cytosol, where it causes X-linked inhibitor of apoptosis protein (XIAP) degradation and the activation of caspases, which lead to apoptosis in cardiomyocytes [26].

A recent study demonstrated the release of HtrA2 into the serum of STEMI patients [18]. If validated in vitro and in vivo, extracellular HtrA2 could have potential as a biomarker for apoptosis; however, in our results necrosis also caused the release of HtrA2.

For cytochrome c, several mechanisms such as the escape of cytochrome c through pores or megachannels, which are formed by proapoptotic Bcl-2 family members, have been proposed for its release into the extracellular space [28,29]. The exact mechanisms of HtrA2′s release into the extracellular space remain to date unknown and need to be investigated in future studies. 

The essential function of HtrA2-inducing intracellular apoptosis has been described intensively in several studies [14,17,26,30]. Cardiac overexpression of HtrA2 in mice induced myocardial apoptosis and led to cardiac dysfunction [17]. Furthermore, preventing HtrA2-induced apoptosis in animal models using a specific HtrA2 inhibitor (UCF-101), resulting in a decrease of myocardial infarct size and better outcome of cardiac contractility function [14].

Altogether, in vitro and in vivo studies have shown the effect of intracellular HtrA2-mediating apoptosis.

Nothing is known about extracellular HtrA2-inducing apoptosis. As a new finding, we detected that extracellular HtrA2 induces apoptosis in HUVECs and that apoptosis can be diminished by adding anti-HtrA2 antibodies. Furthermore, staurosporine-induced apoptosis could be inhibited using anti-HtrA2 antibody. IR is associated with an acute inflammatory response that enhances vascular and tissue damage. As the main focus in this study was to evaluate the effects of extracellular HtrA2, additional experiments were performed in HUVECs to observe whether extracellular HtrA2 has any impact on leukocyte recruiting and activation of the two main inflammatory adhesion molecules intracellular adhesion molecule (ICAM) and vascular adhesion molecule (VCAM). No effect of extracellular HtrA2 was observed (supplemental).

The mechanism of extracellular HtrA2-inducing apoptosis has not been determined. A possible mechanism inducing apoptosis could be the extrinsic apoptotic pathway, cellular uptake or unknown mechanisms. The extrinsic or death-mediated pathway is initiated with the binding of a death ligand such as the tumour necrosis factor alpha or the Fas ligand to its receptor (TNF-α or Fas). This binding leads to the recruitment of a death domain such as the Fas-associated death domain (FADD), which in consequence leads to activation of caspase 8 and its downstream caspases [13]. To confirm this hypothesis further, analysis of activated caspase 8 using western blotting, for instance, is necessary. The role of extracellular HtrA2 might be to influence the extracellular pathway due to its PDZ domain [14]. For serine protease activity, the formation of a homotrimer structure is essential. The Fas ligand also forms a homotrimer, which can assemble into the homotrimeric death receptor Fas [16]. Hence, HtrA2, with its homotrimeric structure, might interact with the homotrimeric death receptors Fas. 

The Fas pathway has already been described to be critical for cardiomyocyte apoptosis and infarct development due to IR [31]. Binding of extracellular HtrA2 could therefore possibly enhance cell death. In our staurosporine apoptosis experiments, extracellular HtrA2 in the cell medium decreased again after four and eight hours, indicating a possible cellular binding of HtrA2 to a receptor or a possible cellular uptake. Future experiments have to assess whether the biphasic release of HtrA2 is due to binding or uptake of the initially released HtrA2, which then again leads to induction of apoptosis, causing a second secretion of new apoptotic cells.

Interestingly, staurosporine-induced apoptosis could be inhibited using anti-HtrA2 antibodies. We hypothesise that the release of HtrA2 after staurosporine induction of apoptosis into the extracellular space might induce a positive loop of HtrA2 inducing apoptosis again. 

**Figure 5:**
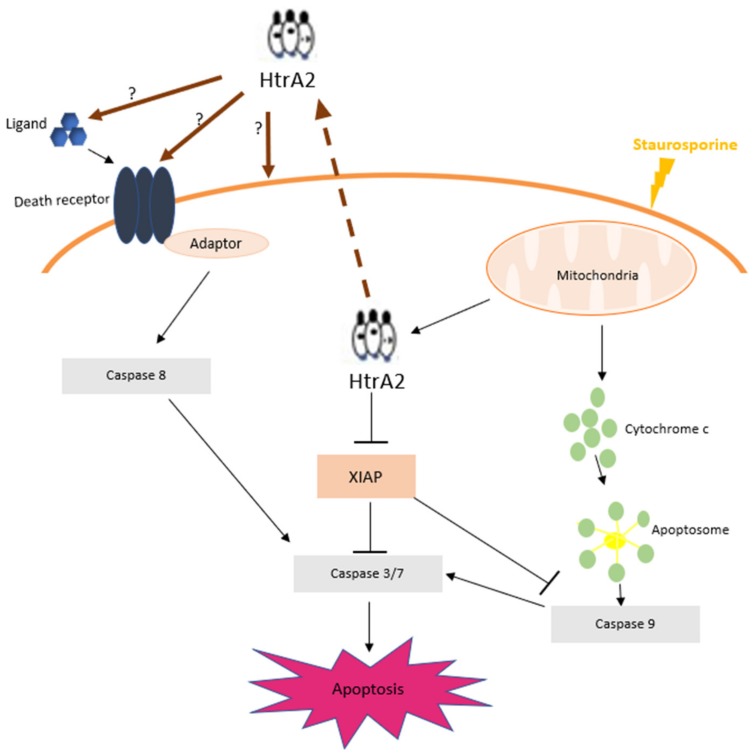
Potential mechanism of HtrA2-mediated release and extracellular induction of apoptosis. This figure further describes already known mechanisms of intracellular induction of apoptosis by HtrA2. The intrinsic apoptotic pathway leads to the release of cytochrome c and HtrA2 from the intermembrane space of mitochondria into the cytosol. Cytochrome c induces apoptosis via caspase activation. HtrA2 proteolyses the inhibitory protein XIAP activating caspase 3/7 and 9 and subsequently induces apoptosis. In our study, apoptosis induction increased extracellular HtrA2 significantly. Extracellular HtrA2 was able to induce apoptosis in HUVEC. A possible mechanism might be the activation of the extrinsic pathway or through unknown mechanism; x-linked inhibitor of apoptosis protein (XIAP). brown arrows = possible mechanism of HtrA2 mediated apoptosis induction, brown dotted arrow = extracellular release of HtrA2, black arrows = activation, black T arrows = inhibition.

The inhibition of extracellular HtrA2-induced apoptosis could be a potential target in cardiac protection, however the underlying mechanism needs to be assessed in future studies. Our new findings are summarised in Figure 5. Furthermore, preventing HtrA2 release in patients with IR injury may decrease infarct size and improve patient outcomes, as already described in animal models [15].

The results of this study support our hypothesis. However, more work is required to understand the potential role of HtrA2 inducing apoptosis. Therefore, this study has some limitations.

Several methods have been developed to detect cell death. As apoptosis/necrosis detection method we used flow cytometry and inverted phase contrast microscopy. The contribution of HtrA2-inducing apoptosis has to be assessed using additional other apoptotic assays, which are based on morphological criteria and distinct markers of apoptotic pathways such as effector caspase activity or DNA fragmentation. Assessing the activation of caspase 3, 7, 8, and 9 will give more insight into the underlying mechanism of HtrA2-induced apoptosis.

Our in vitro study was performed with human umbilical vein endothelial cells. As already demonstrated in several studies, IR contributes to endothelial dysfunction [32,33]. Therefore, HUVECs are being widely used in cardiovascular research as a cell culture model. Experiments should be performed additionally with cardiomyocytes, being the main cells of the heart, damaged through IR injury.

Furthermore, it would be interesting to observe whether HtrA2 is released in a hypoxia-reoxygenation injury cell culture model and whether the damage caused by HtrA2 can be inhibited using anti-HtrA2 antibodies.

The current study provides the necessary preliminary data to further investigate these mechanisms. Still, the pathophysiological substrates for extracellular HtrA2 remain to be determined.

Taken together in the present study, we have demonstrated for the first time that HtrA2 is released extracellularly during apoptosis and that extracellular HtrA2 induces apoptosis in human umbilical vein endothelial cells.

## 4. Materials and Methods

### 4.1. Cell Culture

Human umbilical vein endothelial cells (HUVECs) were isolated from umbilical veins as described before [34]. In brief, 10 cm long umbilical cord veins were cleaned in sterile plastic bags and rinsed with 2 mL Collagenase A at 37 °C (Roche Diagnostics GmbH, Mannheim, Germany). The end of the umbilical cord vein was closed and rinsed again with Collagenase A and incubated at 37 °C. After that, 50 mL of 10% fetal calf serum (FCS) (PELO Biotech, Planegg, Germany) medium was added and centrifuged for 5 min at 300 x g. The cell pellet was resuspended in Endothelial Cell Growth Medium Kit enhanced supplement kit(PELO Biotech, Planegg, Germany), and cell culture flasks were incubated overnight at 37 °C. Endothelial cells were washed twice with Dulbecco′s Phosphate buffered saline with magnesium and calcium (GIBCO^®^ Life Technologies™, Darmstadt, Germany). HUVECs have been characterized by using flow cytometry analysis. For long time, storage cells have been resuspended in recovery^TM^ cell culture freezing medium (containing DMSO) (GIBCO Life Technologies™) and stored in liquid nitrogen.

For each experiment, cells were used at passage 4 and were grown in Endothelial Cell Growth Medium Kit enhanced and supplement kit for Endothelial Cell Growth Medium Kit enhanced (PELO Biotech, Planegg, Germany) factors and 10% FBS at 37 °C in a humidified atmosphere of 5% CO_2_.

### 4.2. Treatment of Cells

HUVECs were cultured in 12 well plates (Thermofischer Scientific, Waltham, MA, USA) at a density of 7 × 10^4^ cells/well and incubated in Endothelial Cell Growth Medium with antibiotics, glutamine, heparin (PELO Biotech, Germany) supplemented with 2,5% FBS. 17–19 h later, the cells were washed and incubated in medium with supplementary inductors; for experiments analyzing the extracellular release of HtrA2, 4 mM H_2_O_2_ (Merck, Darmstadt, Germany) and 200 nM staurosporine (Abcam, Frankfurt, Germany) were used for 24 h.

For experiments analyzing the extracellular effect of HtrA2, 2 µg/mL human recombinant HtrA2 (R&D Systems, Wiesbaden, Germany) +/− 2 µg/mL anti - HtrA2 antibody (R&D systems, Germany) was added to the medium.

After being added, the stimulants cells were incubated at 37 °C in a humidified atmosphere of 5% CO_2_. Controls were added with vehicle. Each treatment was done in triplicate.

### 4.3. HtrA2 in Supernatant of Cell Culture

Culture medium of staurosporine (200nM)- and H_2_O_2_ (4mM)-treated HUVECs were collected after different time points of incubation (2 h, 4 h, 8 h, 12 h and 24 h). After each time point of collection, the medium was centrifuged (500× *g*) and supernatant was collected in a fresh tube and stored at −20°C. The level of HtrA2 in cell culture medium was measured by a commercially available human ELISA immunoassay for HtrA2/Omi (Human HtrA2/Omi Quantikine^®^ ELISA Kit, R&D Systems). The ELISA was performed on samples in duplicate according to the manufacturer′s instructions. All samples and standards were analyzed on a microplate reader set at a wavelength correction of 540 nm. HtrA2 concentrations were interpolated by comparison of the optical densities to the standard curve. The lower detection limit of the assay was 31.25 pg/mL.

### 4.4. Annexin V/PI Staining

The amount of apoptosis was determined as the percentage of Annexin V-positive cells. The amount of necrosis was measured using the percentage of only PI-positive cells.

Annexin-V/PI assays were performed using a commercial apoptosis assay kit (Biolegend, San Diego, CA, USA).

Flow cytometry was performed using Flow Cytometer BD FACS Canto II (Beckton Dickinson, Franklin Lakes, NJ, USA).

### 4.5. Adhesion Molecules Expression on HUVEC

The expression of ICAM (CD54) and VCAM (CD106) was analyzed by flow cytometry using Flow Cytometer BD FACS Canto II (Beckton Dickinson). For this, HUVEC were maintained as mentioned above and were seeded in a 12-well plate at a density of 7 × 10^4^ in Endothelial Cell Growth Medium with antibiotics, glutamine, and heparin (PELO Biotech, Martinsried, Germany) supplemented with 2.5% FBS. 17–19 h later, the cells were washed and treated with 10 ng/mL TNFα (RnD Systems), 2 µg/mL HrA2 and vehicle for 4 h, 8 h and 24 h. After treatment, HUVECs were washed with phosphate buffered saline (PBS) (Thermofischer, Scientific, Waltham, MA, USA), detached with trypsin 0.05% EDTA (Thermofischer, Scientific) and resuspended in 48 µL PBS plus 0.1% bovine serum albumin (BSA) (Merck, Kennelworth, NJ, USA), stained with 1 µL Pacific Blue conjugated antibody CD54 and 1 µL APC conjugated antibody CD106 per sample. After 30 min of incubation at 4 °C, in the dark, the cells were centrifuged at 500 g for 5 min and resuspended in 300 µL PBS plus 0.1% BSA.

### 4.6. Morphologic Observation

HUVECs were cultured in 12 well plates (Thermofischer Scientific, USA) at a density of 4 × 10^4^ cells/well and incubated in Endothelial Cell Growth Medium with antibiotics, glutamine, and heparin (PELO Biotech) supplemented with 2.5% FBS. 17-19 h later, the cells were washed and incubated in medium with 4 mM H_2_O_2_ (Merck, Darmstadt, Germany), 200 nM staurosporine (Abcam), 2 µg/mL HrA2 and vehicle for 2 and 24h. The morphology of HUVECs was observed under an inverted phase contrast microscope (microscope: AXIO Vert.A1, Zeiss, camera: AxioCam ICc1).

### 4.7. Statistics

All values in the text and figures are presented as means ±SD of at least two independent experiments performed in triplicates. Significant differences during time course experiments between groups were performed by using two-way Anova followed by post-hoc Bonferroni test. When we determined the endothelial damage, at an individual time point, Students *t*- test was used. Significance was accepted at *p* < 0.05. All analyses were performed using Graph Pad Prism 8 (GraphPad Software, Inc., La Jolla, CA, USA).

## Abbreviations

ATP	Adenosine tri phosphate
IR	Ischemia reperfusion
TNF	Tumor necrosis factor
HtrA2	High temperature required protein A2
STEMI	ST-Elevation myocardial infarction
Bcl	B-cell lymphoma
FADD	Fas-associated protein with death domain
VCAM	Vascular adhesion molecule
ICAM	Intracellular adhesion molecule
PI	Propidium iodide
PDZ	PSD-95, discs large, zona occludens 1
XIAP	X-linked Inhibitor of apoptosis protein
PBS	Phosphate buffered saline
BSA	Bovine serum albumin
S.D	Standard Deviation

## References

[1] Https://www.Who.Int/news-room/fact-sheets/detail/the-top-10-causes-of-death.

[2] Davie A.P. (1996). Early thrombolytic treatment in acute myocardial infarction. Lancet.

[3] Hausenloy D.J., Yellon D.M. (2013). Myocardial ischemia-reperfusion injury: A neglected therapeutic target. J. Clin. Invest..

[4] Eltzschig H.K., Eckle T. (2011). Ischemia and reperfusion--from mechanism to translation. Nat. Med..

[5] Frank A., Bonney M., Bonney S., Weitzel L., Koeppen M., Eckle T. (2012). Myocardial ischemia reperfusion injury: From basic science to clinical bedside. Semin. Cardiothorac Vasc. Anesth..

[6] Wu M.Y., Yiang G.-T., Liao W.-T., Tsai A.P.-Y., Cheng Y.-L., Cheng P.-W., Li C.-Y., Li C.-J. (2018). Current mechanistic concepts in ischemia and reperfusion injury. Cell. Physiol. Biochem..

[7] Kalogeris T., Baines C.P., Krenz M., Korthuis R.J. (2016). Ischemia/reperfusion. Compr. Physiol..

[8] Kalogeris T., Baines C.P., Krenz M., Korthuis R.J. (2012). Cell biology of ischemia/reperfusion injury. Int. Rev. Cell Mol. Biol..

[9] Hausenloy D.J., Yellon D.M. (2003). The mitochondrial permeability transition pore: Its fundamental role in mediating cell death during ischaemia and reperfusion. J. Mol. Cell Cardiol..

[10] Whelan R.S., Kaplinskiy V., Kitsis R.N. (2010). Cell death in the pathogenesis of heart disease: Mechanisms and significance. Annu. Rev. Physiol..

[11] Konstantinidis K., Whelan R.S., Kitsis R.N. (2012). Mechanisms of cell death in heart disease. Arterioscler. Thromb. Vasc. Biol..

[12] Hashmi S., Al-Salam S. (2015). Acute myocardial infarction and myocardial ischemia-reperfusion injury: A comparison. Int. J. Clin. Exp. Pathol..

[13] Elmore S. (2007). Apoptosis: A review of programmed cell death. Toxicol. Pathol..

[14] Bhuiyan M.S., Fukunaga K. (2008). Activation of htra2, a mitochondrial serine protease mediates apoptosis: Current knowledge on htra2 mediated myocardial ischemia/reperfusion injury. Cardiovasc. Ther..

[15] Bhuiyan M.S., Fukunaga K. (2007). Inhibition of htra2/omi ameliorates heart dysfunction following ischemia/reperfusion injury in rat heart in vivo. Eur. J. Pharmacol..

[16] Wang K., Zhang J., Liu J., Tian J., Wu Y., Wang X., Quan L., Xu H., Wang W., Liu H. (2013). Variations in the protein level of omi/htra2 in the heart of aged rats may contribute to the increased susceptibility of cardiomyocytes to ischemia/reperfusion injury and cell death: Omi/htra2 and aged heart injury. Age.

[17] Wang K., Yuan Y., Liu X., Lau W.B., Zuo L., Wang X., Ma L., Jiao K., Shang J., Wang W. (2016). Cardiac specific overexpression of mitochondrial omi/htra2 induces myocardial apoptosis and cardiac dysfunction. Sci. Rep..

[18] Hortmann M., Robinson S., Mohr M., Haenel D., Mauler M., Stallmann D., Reinoehl J., Duerschmied D., Peter K., Bode C. (2017). Circulating htra2 as a novel biomarker for mitochondrial induced cardiomyocyte apoptosis and ischemia-reperfusion injury in st-segment elevation myocardial infarction. Int. J. Cardiol..

[19] Renz A., Berdel W.E., Kreuter M., Belka C., Schulze-Osthoff K., Los M. (2001). Rapid extracellular release of cytochrome c is specific for apoptosis and marks cell death in vivo. Blood.

[20] Ahlemeyer B., Klumpp S., Krieglstein J. (2002). Release of cytochrome c into the extracellular space contributes to neuronal apoptosis induced by staurosporine. Brain Res..

[21] Jemmerson R., LaPlante B., Treeful A. (2002). Release of intact, monomeric cytochrome c from apoptotic and necrotic cells. Cell Death Differ..

[22] Lee Y., Gustafsson A.B. (2009). Role of apoptosis in cardiovascular disease. Apoptosis.

[23] Eefting F., Rensing B., Wigman J., Pannekoek W.J., Liu W.M., Cramer M.J., Lips D.J., Doevendans P.A. (2004). Role of apoptosis in reperfusion injury. Cardiovasc Res..

[24] Heusch G., Gersh B.J. (2017). The pathophysiology of acute myocardial infarction and strategies of protection beyond reperfusion: A continual challenge. Eur. Heart J..

[25] Claeys M.J., Bosmans J., Veenstra L., Jorens P., De Raedt H., Vrints C.J. (1999). Determinants and prognostic implications of persistent st-segment elevation after primary angioplasty for acute myocardial infarction: Importance of microvascular reperfusion injury on clinical outcome. Circulation.

[26] Liu H.R., Gao E., Hu A., Tao L., Qu Y., Most P., Koch W.J., Christopher T.A., Lopez B.L., Alnemri E.S. (2005). Role of omi/htra2 in apoptotic cell death after myocardial ischemia and reperfusion. Circulation.

[27] Hausenloy D.J., Garcia-Dorado D., Botker H.E., Davidson S.M., Downey J., Engel F.B., Jennings R., Lecour S., Leor J., Madonna R. (2017). Novel targets and future strategies for acute cardioprotection: Position paper of the european society of cardiology working group on cellular biology of the heart. Cardiovasc. Res..

[28] Desagher S., Osen-Sand A., Nichols A., Eskes R., Montessui S., Lauper S., Maundrell K. (1999). Bid-induced conformational change of bax is responsible for mitochondrial cytochrome c release during apoptosis. J. Cell Biol..

[29] Eskes R., Desagher S., Antonsson B., Martinou J.C. (2000). Bid induces the oligomerization and insertion of bax into the outer mitochondrial membrane. Mol. Cell Biol..

[30] Marabese M., Mazzoletti M., Vikhanskaya F., Broggini M. (2008). Htra2 enhances the apoptotic functions of p73 on bax. Cell Death Differ..

[31] Lee P., Sata M., Lefer D.J., Factor S.M., Walsh K., Kitsis R.N. (2003). Fas pathway is a critical mediator of cardiac myocyte death and mi during ischemia–reperfusion in vivo. Am. J. Physiol. Heart Circ. Physiol..

[32] Yang Q., He G.W., Underwood M.J., Yu C.M. (2016). Cellular and molecular mechanisms of endothelial ischemia/reperfusion injury: Perspectives and implications for postischemic myocardial protection. Am. J. Transl. Res..

[33] Singhal A.K., Symons J.D., Boudina S., Jaishy B., Shiu Y.T. (2010). Role of endothelial cells in myocardial ischemia-reperfusion injury. Vasc. Dis. Prev..

[34] Hornstein A.M. (2016). Effects of specific bone morphogenetic proteins on endothelial permeability. Mensch und Buch Verl..

